# O^6^-methylguanine-DNA methyltransferase in equine sarcoids: molecular and epigenetic analysis

**DOI:** 10.1186/1746-6148-8-218

**Published:** 2012-11-10

**Authors:** Gennaro Altamura, Maria Strazzullo, Annunziata Corteggio, Romina Francioso, Franco Roperto, Maurizio D'Esposito, Giuseppe Borzacchiello

**Affiliations:** 1 Department of Pathology and Animal health, University of Naples Federico II, Via Veterinaria, 1-80137, Napoli, Italy; 2 Institute for Animal Production System in Mediterranean Environment, National Research Council, Via Argine, 1085 80147, Naples, Italy; 3 Institute of Genetic and Biophysics ABT, National Research Council, Via P.Castellino 111, 80131, Naples, Italy; 4 IRCCS Neuromed, Pozzilli, Italy

**Keywords:** BPV, Equine sarcoid, MGMT

## Abstract

**Background:**

Bovine papillomaviruses (BPVs) types 1 and 2 are the only known papillomaviruses able to jump the species. In fact, BPVs 1/2 induce neoplasia in their natural bovine host but infection is also associated to neoplastic skin lesions in equids termed sarcoids. The equine sarcoid is considered to be the most common equine cutaneous tumour worldwide for which no effective therapy is available. Very little is known about the molecular mechanisms underlying tumourigenesis, although genes contributing to sarcoid development have been identified. Several studies associate the development of cancer to the loss of function of a number of oncosuppressor genes. In this study the putative role of O^6^-methylguanine-DNA methyltrasferase (MGMT) was investigated for sarcoids. The expression of the oncosuppressor protein was assessed in normal and sarcoid cells and tissues. In addition, the DNA methylation profile was analysed to assess the role of epigenetic mechanism in regulation of MGMT expression.

**Results:**

A group of 15 equine sarcoids and two primary sarcoid cell lines (fibroblasts) were analyzed for the expression of MGMT protein by immunohistochemistry, immunofluorescence and Western blotting techniques. The sarcoid cell line EqSO4b and the tumour samples showed a reduction or absence of MGMT expression. To investigate the causes of deregulated MGMT expression, ten samples were analyzed for the DNA methylation profile of the CpG island associated to the MGMT promoter. The analysis of 73 CpGs encompassing the region of interest showed in 1 out of 10 (10%) sarcoids a pronouncedly altered methylation profile when compared to the control epidermal sample. Similarily the EqSO4b cell line showed an altered MGMT methylation pattern in comparison to normal fibroblasts.

**Conclusion:**

As previously demonstrated for the oncosuppressor gene FHIT, analysis of MGMT expression in sarcoid tissues and a sarcoid-derived fibroblast cell line further suggests that oncosuppressor silencing may be also involved in BPV-induced equine tumours. Abnormal DNA methylation seems to be one of the possible molecular mechanisms involved in the alteration of MGMT expression. Further studies are required to address other basic molecular mechanisms involved in reduced MGMT expression. This study underlines the possible role of DNA methylation in oncosuppressor inactivation in equine sarcoids.

## Background

Sarcoids are locally invasive skin tumours of equids and are considered to be the most common equine cutaneous neoplasm worldwide [1]. Macroscopically, sarcoids vary and six different clinical types are recognized: occult, verrucous, nodular, fibroblastic, mixed and malevolent [2]. They are histologically characterized by disorganized dermal proliferation of spindle-shaped fibroblasts that form whorls and by epidermal hyperplasia, hyperkeratosis, and rete peg formation [3]. The tumours can occur anywhere on animal’s body; however they often arise in areas of previous injury or scarring; additionally, they very rarely regress, more often persist and can be locally aggressive. Currently, there is no effective therapy available for sarcoids [4].

Many reports in the literature have shown that BPV type 1 and less commonly type 2 are involved in the pathogenesis of sarcoids. Recent studies have highlighted the role of the BPV oncogenes E5 and E7 in the carcinogenesis [5-7]. However, little is known regarding the molecular mechanisms underlying sarcoid tumourigenesis and only few genes contributing to the development of this neoplastic disease have been identified so far [8].

Several studies associate the development of cancer to the loss of function of a number of oncosuppressor genes. In a recent work (Strazzullo et al., 2012) we have provided evidence for an altered expression of the FHIT protein in sarcoid tissues and derived cell lines. FHIT is the first oncosuppressor gene whose aberrant expression has been associated to equine sarcoids whereas it is frequently altered in human tumours associated to papillomavirus infection.

MGMT is another oncosuppressor gene often inactivated in human PV-induced tumours. This gene, also called O^6^-alkylguanine-DNA alkyltransferase, encodes for a DNA repair protein that removes mutagenic and cytotoxic adducts from O^6^-guanine in DNA [9]. Alkylation of DNA at the O^6^ position of guanine is an important step in the formation of mutations in cancer, primarily due to the tendency of the O^6^–methylguanine to pair with thymine during replication, resulting in the conversion of guanine-cytosine to adenine-thymine pairs in DNA [10]. Moreover, the O^6^ –methylguanine adducts crosslink with the opposite cytosine residues, blocking DNA replication [11]. MGMT is able to protect cells from such biological processes, resulting in the direct restoration of the normal guanine structure [12]. The alkylated MGMT becomes detached from DNA and is degraded through ubiquitination-dependent proteolysis [13].

The expression of the MGMT protein is decreased in a wide spectrum of human tumours due to several genetic mechanisms and as a consequence of the hypermethylation of the promoter region [14]. In some human cancers, such as gliomas, inactivation of MGMT gene correlates with a better prognosis as this condition allows a better responsiveness to therapy with alkylating agents [15].

MGMT is a well-known target for methylation inactivation in HPV-induced cancer [16] but its role in BPV-induced cancer has not been investigated so far. DNA methylation is a frequent epigenetic event in many human cancers. Many studies have pointed out that promoter methylation of tumour suppressor genes is linked with HPV-induced cervical carcinogenesis [16,17].

Whilst alterations in MGMT expression have been reported for several human cancers comprising those induced by HPV, a putative role of this gene and of epigenetic alteration, to the best of our knowledge, has never been investigated in veterinary oncology [14,15,18].

In order to gain new insights into possible mechanisms underlying BPV-mediated carcinogenesis, we investigated the status of MGMT protein expression in equine sarcoid cell lines and sarcoid tumours. The samples showing significantly reduced MGMT protein expression were further analyzed for the DNA methylation status of equine MGMT CpG island spanning the predicted 5^′^UTR.

## Results

### MGMT protein expression in sarcoids and cell lines

**Figure 1:**
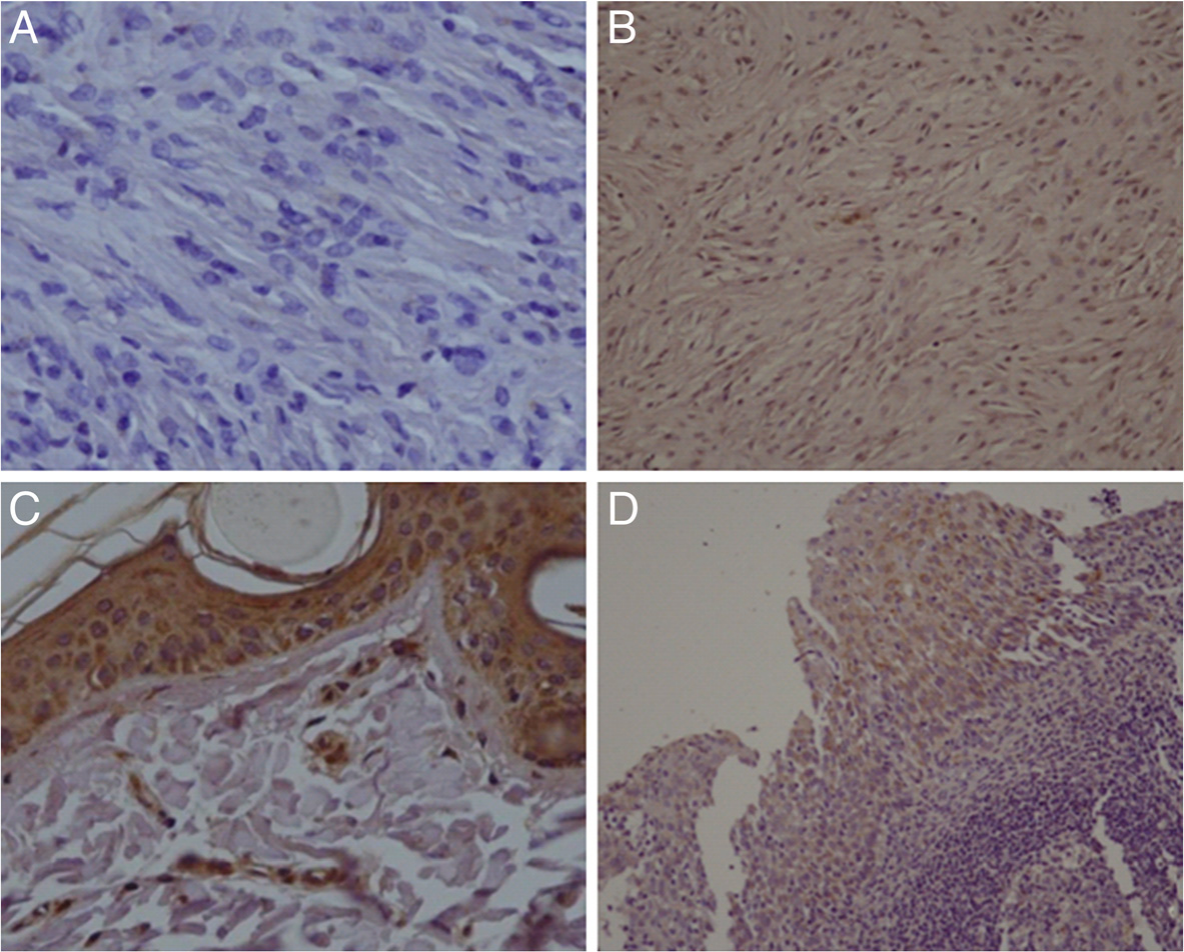
**Illustration of representative immunohistochemistry in equine normal skin and sarcoid lesions.A**) MGMT negative immunoreactivity in a sarcoid sample. Neoplastic fibroblasts are negative for MGMT staining. Strepatavidin-biotin peroxidase method. Mayer’s haematoxylin nuclear counterstaining. x 240. **B**) Sarcoid sample showing very weak MGMT immunoreactivity. Some fibroblasts from the neoplastic tissue show a very faint cytoplasmic immunostaining for MGMT. Strepatavidin-biotin peroxidase method. Mayer’s haematoxylin nuclear counterstaining. IHC. x 240. **C**) MGMT staining of normal equine skin. Normal fibroblasts from derma displayed nuclear and cytoplasmic staining for MGMT. The different epidermal layers are also stained. Strepatavidin-biotin peroxidase method. Mayer’s haematoxylin nuclear counterstaining. IHC. x 240. **D**) Human tonsil tissue section. Epithelial cells show positive MGMT immunostaining. Strepatavidin-biotin peroxidase method. Mayer’s haematoxylin nuclear counterstaining. IHC. x 120.

To investigate the potential oncosuppressor involvement in equine sarcoid carcinogenesis, we analyzed immunohistochemically 10 out of 15 tumour samples for MGMT expression. Negative staining for MGMT protein was observed in 5 out of 10 cases (50%) (Figure 1A); 2 sarcoid samples (20%) displayed very weak immunostaining signal in only few areas of the lesion (data not shown). The remaining sarcoid samples (30%), showed a very faint intracytoplasmic immunostaining signal for MGMT throughout the lesion (Figure 1B).

Normal fibroblasts derived from healthy horses displayed nuclear and cytoplasmic staining pattern for the MGMT protein. Additionally, cells from the different skin layers also stained positive (Figure 1C). Human tonsil tissue section used as positive control showed positive immunostaining (Figure 1D).

**Figure 2:**
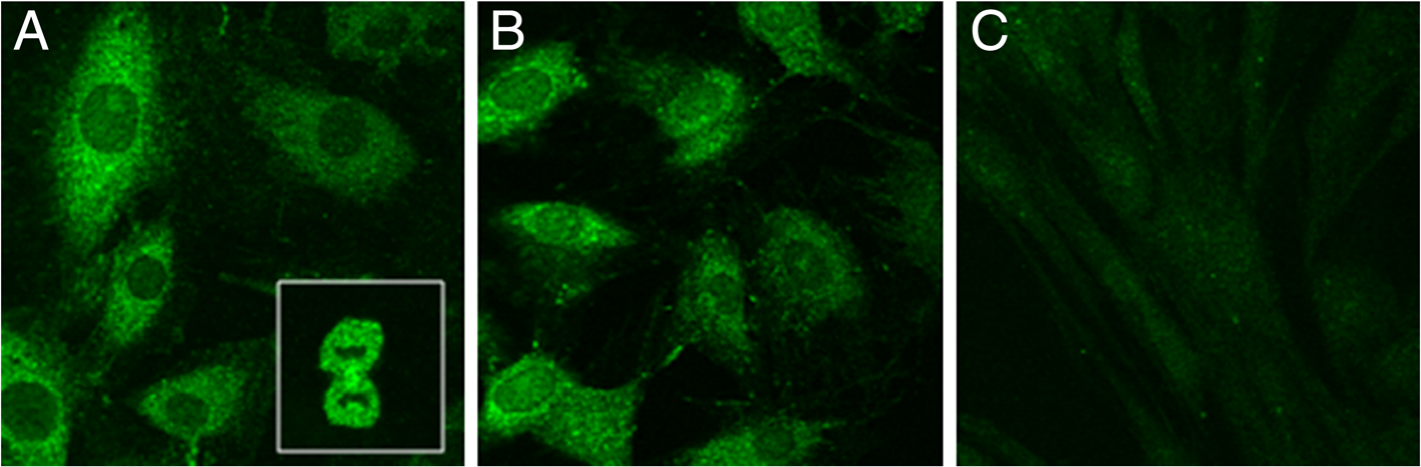
**Down-regulated MGMT protein expression in equine sarcoidfibroblasts.** Immunofluorescence-mediated detection of MGMT protein in normal equine fibroblasts and sarcoid fibroblasts, assessed by confocal microscopy. **A**) Normal fibroblasts cell line E-Derm shows cytoplasmic staining for MGMT. Nuclei of mitotic cells express MGMT (insert).x120. **B**) Fully transformed fibroblasts EqSO1a show a faint cytoplasmic staining. x120. **C**) Fully transformed fibroblasts EqSO4b show a very faint cytoplasmic staining. x120.

We also analyzed the equine sarcoid derived cell lines for the expression of MGMT protein. By indirect immunofluorescence, distinct staining was detected in the E-Derm and EqSO1a sarcoid cell lines. In E-Derm the staining pattern was diffuse and the immunofluorecence signal was detected within the cytosol as well as in the nuclei of mitotic cells (Figure 2A). In EqSO1a the immunofluorescence signal was detected only within the cytosol and the staining pattern was diffuse (Figure 2B). IF staining of the sarcoid cell line EqSO4b yielded very weak and hardly perceptible signal for MGMT protein (Figure 2C).

**Figure 3:**
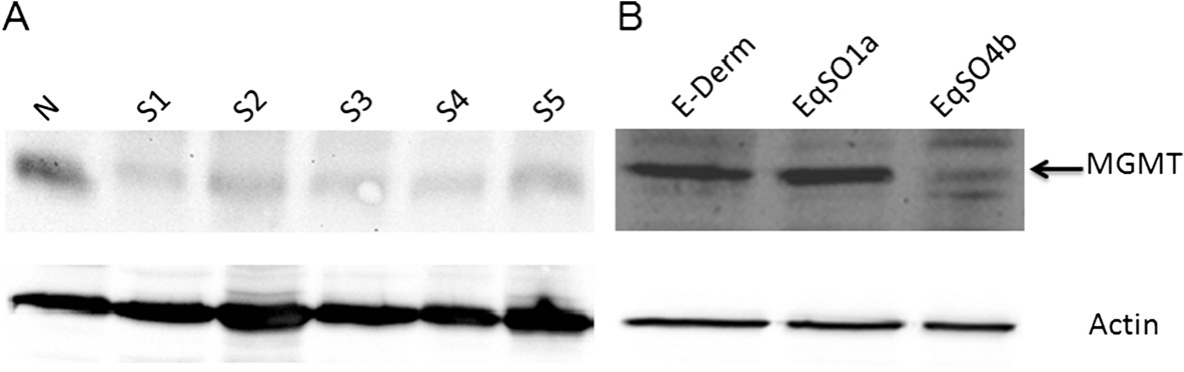
**MGMT molecular expression in sarcoid tissues and sarcoid derived cell lines.A**) Western blotting showing the reduced protein expression of MGMT in sarcoid tissues (S1-S5) in comparison with a normal skin sample (N). **B**) Western blotting showing the reduced *in vitro* protein expression of MGMT in 2 sarcoid cell lines (EqSO1a, EqSO4b) in comparison with normal fibroblast cell line (E-Derm). The lower blot shows actin expression to demonstrate the same amount of protein loaded onto the gel.

To further confirm our finding of reduced intralesional MGMT protein expression, 5 sarcoid samples, which were available for biochemical analysis and skin from a healthy horse were analysed by Western blot. Cell lines were also analyzed. The anti-MGMT antibody yielded a band of the expected molecular weight in the neoplastic tissues, normal skin and all fibroblast cell lines. An increase in the amount of MGMT protein level in normal skin versus tumour samples was observed (Figure 3A). In addition, MGMT expression levels were lower in EqSO4b sarcoid cell line, whilst EqSO1a showed similar MGMT expression when compared to E-Derm cells (Figure 3B).

### Equine MGMT CpG island and DNA methylation analysis in sarcoid tissues and cell lines

The 10 sarcoids that stained biochemically or immunohistochemically negative or very weakly positive for MGMT protein expression, were analyzed for the potential involvement of epigenetic mechanisms in the abnormal expression of this oncosuppressor. To this aim, DNA methylation profile of the CpG island associated to the putative regulatory region was analyzed. A similar analysis was performed also for sarcoid-derived cell lines EqSO1a and EqSO4b.

**Figure 4:**
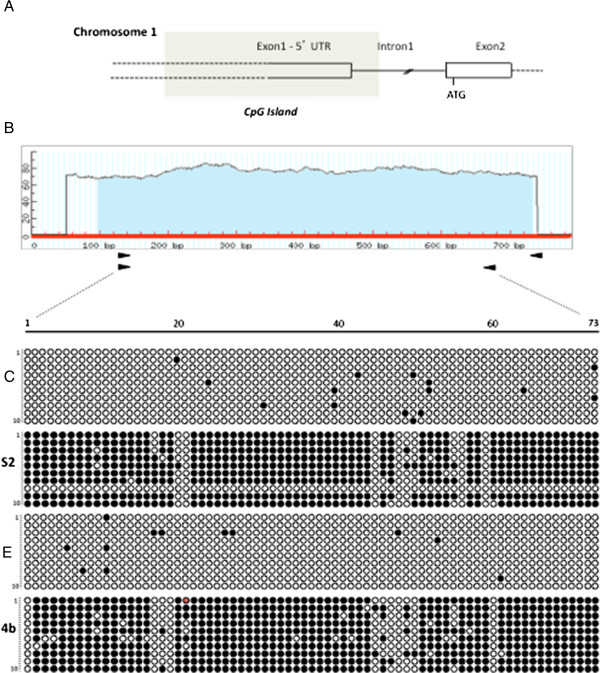
**Horse MGMT locus organization and schematic representation of the sodium bisulfite analysis.A**) A mere schematic, not in scale, presentation of the 5'UTR of the MGMT genomic locus on chromosome 1 is shown; the shaded box indicates the position of the CpG island with respect to the exon 1 and exon 2. The ATG position is also reported. The dotted line indicates the not defined boundary between Exon 1 and regulative region. **B**) In the top are summerised the Methprimer predicted CpG island and the position of primers used for nested PCR. In the lower part, circles indicate methylation status: filled circles, methylated CpGs; open circles: unmethylated CpGs. Each row of circles corresponds to one clone. For each sample 73 CpGs were analysed; the column on the left of each sample are referred to the number of indipendent clones sequenced. **C**, control tissue; **S2**, sarcoid sample; **E**, E-Derm cell line; **4b**, Sarcoid cell line EqSO4b.

The only equine MGMT sequence available in GenBank, is the predicted coding sequence and a partial 5^′^ UTR region [XM_001488425.3]. To obtain the horse specific sequence of the upstream region corresponding to the human, murine and bovine CpG island, the cds sequence was used to query the UCSC database. Through the BLAT algorithm and using the Horse Sep. 2007 (Broad/equCab2) Assembly, the MGMT upstream region was obtained (about 800 bp). An unknown region we localized in the UCSC sequence was identified by PCR amplification and subsequent bidirectional sequencing (see material and methods). The CpGplot analysis revealed a CpG island of about 635 bp. A region of 573 bp, including 73 CpGs, was subjected to sodium bisulfite sequencing. Figure 4A shows a not in scale scheme of the 5^′^ region of the equine MGMT locus, and the relative position of the predicted CpG island (shaded) as reconstructed according to the information obtained from UCSC and NCBI databases.

The DNA extracted from the sarcoid samples and from a BPV negative normal skin sample, was converted by sodium bisulfite. Figure 4B on the top, shows the CpG island prediction obtained through the MethPrimer algorithm ( http://www.urogene.org/methprimer/index1.html) and the position of the specific primers obtained using the same algorithm. The region of interest was amplified, cloned and sequenced. For each samples at least 10 clones were sequenced. One sarcoid sample showed a clear difference in the DNA methylation content compared to the normal control. A similar analysis was performed also for the sarcoid cell lines, EqSO1a and EqSO4b compared to the E-Derm fibroblast cell lines. Also the EqSO4b showed a different methylation pattern when compared to the normal skin derived cell line E-Derm, similarly to that observed in the positive sarcoid sample. In the lower part of Figure 4B, the position of methylated and unmethylated cytosines in the different clones sequenced is shown for normal and sarcoid tissue and for the E-Derm cell line and sarcoid cell line EqSO4b.

## Discussion

DNA methylation is a frequent epigenetic event in many human cancers [19,20]. The transcriptional silencing by the hypermethylation of CpG islands in the promoter region, is a well-known common mechanism for the inactivation of tumour suppressor genes in human malignancies [21,22]. In an ongoing study aiming at investigating the possible epigenetic changes of oncosuppressor genes involved in sarcoid carcinogenesis, we have recently demonstrated a noticeable alteration of expression of the FHIT protein (Strazzullo et al., 2012), which encouraged the investigation of the role of other oncosuppressor genes and of their regulative mechanisms in sarcoid molecular pathology. Since many lines of evidence support the association between HPV infection and MGMT expression alteration [23,24], we analyzed the role of the MGMT gene.

In this study we investigated the expression of the MGMT protein within a normal fibroblast cell line (E-Derm) and the fully transformed sarcoid fibroblast lines explanted from an equine sarcoid tumour (EqSO1a and EqSO4b), as well as the pattern of expression in normal skin and sarcoid tissues. Our data indicate, for the first time, a noticeable reduction and or absence of MGMT protein in naturally occurring sarcoid tumours as well as in sarcoid derived cell lines.

Sarcoids may exist as six different clinical types [2], we have not found a correlation between down-regulation of MGMT expression and clinical appearance, suggesting the existence of a common mechanism underlying the reduction of protein expression that acts early during the development of equine sarcoids. Our results are in agreement with several previous studies indicating that the loss and or reduced expression of MGMT is frequent in a variety of tumours [25], particularly in HPV-induced cervical lesions [26-28]. It is possible that decrease of MGMT protein expression is a common mechanism of cancer development among different species.

It has been suggested that the activity of MGMT increases as the severity of neoplasia and its clinical stage also increases [29,30]. Sarcoid is considered as a “benign” non metastatising tumour. In this regard, it is reasonable to speculate that the reduction and/or absence of MGMT in sarcoids may be correlated to their relative benign biological behavior [1,4].

The presence of a functional MGMT gene is essential to avoid occurring mutations in other important genes involved in cancerogenic processeses. The silencing of MGMT gene induces a mutator pathway affecting some oncogenes such as Ras [31]. It is worth noting that sarcoid tumours and sarcoid derived cell lines show an up-regulation of Ras activity (Altamura et al., manuscript in preparation). It is therefore possible to hypothesize a link between MGMT down-regulation and Ras constitutive activation. However, further investigations are needed to confirm this, which is at moment only a matter of speculation.

As previously observed for the FHIT protein, EqSO4b cells revealed lower MGMT expression in comparison to EqSO1a. MGMT reduced expression in vitro has been linked to its binding to HPV E6 oncoprotein which is able to enhance the ubiquitin-dependent proteolysis of this oncosuppressor [13]. Additionally, HPV-16 E7 is able to modulate the DNA methylation activity [32]. Since the EqSO4b cell line contains higher copy numbers of viral genome and oncoprotein transcripts than the EqSO1a [33], it is possible that the observed decreased level of MGMT protein found may directly correlate with BPV oncogene expression levels and be due to a similar mechanisms induced by BPV E5/E7 oncoproteins. Further studies are needed to elucidate the molecular mechanisms underlying this peculiar aspect.

Promoter methylation is the primary epigenetic alteration associated with transcriptional silencing of tumour suppressor genes during tumourigenesis [34-36]. To determine if DNA methylation was involved in altered MGMT expression in sarcoid tumours, we performed the sodium bisulfite sequencing analysis of the CpG island associated to the 5^′^ untranslated region of MGMT [37]. Our data indicated that in MGMT gene promoter heavy hypermethylation occurred in 1 out of the 10 analyzed sarcoid samples and in EqSO4b cells. The sarcoid sample and the EqSO4b cell line showed an overall similar hypermethylation pattern. Differences may be explained by the presence of some polymorphisms, a fortiori considering that the analysis was carried out for a not translated region. EqSO4b is a primary culture cell line and this may introduce some difference too. Moreover it’s to be considered that the region is very complex in its sequence structure and this may promote polymerase slippage.

Similar frequency of MGMT hypermethylation is observed in HPV induced cervical cancer [38], thus supporting the hypothesis that MGMT promoter methylation is not a common feature of PVs induced tumours.

Our findings indicate an association between BPV infection and MGMT protein expression alteration suggesting the possibility of a mechanistic role for this gene as a cofactor triggering the development of equine sarcoid tumour in concert with BPV.

## Conclusions

The biochemical, immunohistochemical and immunofluorescence analyses suggest that MGMT protein is reduced or absent in sarcoid tissues and sarcoid derived cell lines. The epigenetic analysis suggests DNA methylation as one of the possible causative mechanisms for the loss of expression of MGMT in this tumour. Further studies will be useful to confirm these findings and to clarify the other mechanisms underlying MGMT alteration in sarcoid carcinogenesis, but this study represents the first example in veterinary oncology of the epigenetic inactivation of the MGMT oncosuppressor.

## Methods

### Tumour samples

Samples of equine sarcoid and normal skin from healthy horse (N°15) were derived from the archives of the Department of Pathology and Animal Health, University of Naples “Federico II”. Sections taken from paraffin blocks were stained by haematoxylin and eosin (HE) and re-evaluated to confirm the diagnosis. Histologically, the samples were characterized by epidermal hyperplasia with rete peg invading the dermal tissue beneath. A diffuse proliferation of dermal fibroblasts arranged in whorls and or bundles was seen. A storiform pattern was also recorded. All the tumour samples were diagnosed as equine sarcoids.

The samples were derived from different animals. All sarcoids were known to be positive for BPV DNA (Borzacchiello et al., 2008).

Five out of 15 sarcoids were immediately frozen at −80°C and available for biochemical analysis.

### Cell cultures

E-Derm fibroblast cell lines, derived from horse dermis, were obtained from the American Type Culture Collection. EqSO1a and EqSO4b have been described previously (Yuan et al., 2008). All cells were maintained in culture in DMEM (Dulbecco's modified eagle medium) supplemented with 10% FBS (Gibco) in a 37°C humidified atmosphere of 5% CO2 in air.

### Immunohistochemistry

Ten sarcoid samples and 1 skin sample were stained. A section from a normal human tonsil was included as positive control [39]. Briefly, paraffin sections were deparaffinized, and blocked for endogenous peroxidase in 0.3% H_2_O_2_ in methanol for 20 min. Antigen enhancement was performed by pretreating with microwave heating (twice for 5 min each at 525 W). The anti-MGMT antibody (Resnova, Rome, Italy) was applied at 1:50 dilution overnight at room temperature in a humified chamber. The slides were washed three times with phosphate-buffered saline (PBS), then incubated for 30 min with the appropriate biotinylated secondary antibody (labelledstreptavidin–biotin (LSAB) Kit; DakoCytomation, Denmark) as previously reported (Borzacchiello et al., 2006 Oncogene). Sections were washed three times with PBS and then incubated with streptavidin-conjugated to horseradish peroxidase (LSAB Kit; DakoCytomation, Denmark). Colour development was obtained by treatment with diaminobenzidine (DakoCytomation, Denmark) for 5 min. Sections were counterstained with Mayer’s haematoxylin. In the corresponding negative control section, the primary antibody was either omitted or replaced with appropriate normal serum.

The scoring of the immunoreactivity was determined in a ‘blind’ study by two observers (GB and AC). The intensity of labelling in each specimen was scored from absent to very strong immunosignal.

### Immunofluorescence and confocal laser-scanning microscopy

E-Derm, EqSO1a and EqSO4b cell lines were grown for 2 days on coverslips, washed with PBS, fixed in 4% paraformaldehyde for 20 min, permeabilized with 0,1% triton X-100 in PBS 5 min. The slides were blocked with 2% BSA for 30 min. The anti-MGMT primary antibody was applied O/N at 4°C in a humified chamber at 1:50 dilution and after washing with PBS, incubated with Alexa Fluor 488 goat anti-rabbit 30 min at RT (Molecular Probes. Leiden, The Netherlands).

Finally, after washing with PBS, the slides were mounted in aqueous medium PBS: Glycerol 1:1 (Sigma, Milan, Italy). For scanning and photography, a confocal laser-scanning microscope LSM-510 (Zeiss, Gottingen, Germany) was used. The lambda of the argon ion laser was set at 488 nm, and fluorescence emission was revealed by BP 505–530 nm band pass filter.

### Protein extraction and SDS PAGE/western blotting

Five sarcoids (S1;S2;S3;S4;S5) and one sample of normal skin (N) were available for molecular analysis. These were snap frozen in liquid nitrogen and homogenized in ice-cold lysis buffer (50mM Tris pH7.5; 150mM NaCl; 1mM EDTA; 0.25% Deoxicolic acid, 1% Triton X100) added with 20 mM sodium pyrophosphate, 0.1 mg/ml aprotinin, 2 mM phenylmethylsulphony fluoride (PMSF), 10 mM sodium orthovanadate (Na2VO3), and 50 mM NaF.

Cell lines were grown for 2 days in 60-mm dishes, washed with ice-cold phosphate saline buffer two times and lysed for 20 minutes in ice-cold lysis buffer. Tissue homogenates and cell lysates were clarified by centrifugation, and the quantity of proteins was determined by use of a protein assay kit (Bio-Rad Laboratories, Milan, Italy). 50 μg of total protein were boiled and fractionated in 15% SDS-PAGE gel. The proteins were blotted from the gel onto nitrocellulose membranes. The membranes were blocked with 5% non-fat dry milk in TBS buffer at room temperature, washed with TBS–0.1% Tween and incubated with anti-MGMT antibody at dilution 1: 500. After appropriate washing steps, peroxidase-conjugated anti-rabbit IgG (Amersham Pharmacia Biotech) were applied for 1 hour at 1:5,000. After washing, bound antibody was visualized on ECL film (Amersham Pharmacia Biotech). The blots were stripped and reprobed against mouse anti-actin antibody (Calbiochem) at 1:5,000 to confirm equal loading of proteins in each lane.

### Bioinformatic web tools for sequence analyses and primer design

NCBI Genbank ( http://www.ncbi.nlm.nih.gov/sites/entrez) to obtain gene specific sequences; UCSC BLAT ( http://genome.ucsc.edu/cgi-bin/hgBlat) to obtain the sequence of non-coding regions and to recovery and compare the sequences of different species. BLAST algorithm ( http://blast.ncbi.nlm.nih.gov/Blast.cgi) to identify the more informative sequences, to compare the horse specific nucleotidic sequences with that of other species; Primer3plus ( http://www.bioinformatics.nl/cgi-bin/primer3plus/primer3plus.cgi) and NCBI primer-blast ( http://www.ncbi.nlm.nih.gov/tools/primer-blast/index.cgi) to design PCR primers; EMBOSS CpGPlot/CpGReport/Isochore ( http://www.ebi.ac.uk/Tools/emboss/cpgplot/) for the analysis of the CpG content.

### Cell line DNA extraction and CpG island gap sequencing

Genomic DNA was extracted from one 60-mm dish respectively of E-Derm, EqSO1a and EqSO4b cell lines according to the Wizard Genomic DNA Purification Kit protocol (Promega, corporation, USA). The purified DNA was resuspended in TE.

The lacking sequence of the equine specific MGMT CpG island was obtained by PCR amplification using as template E-Derm genomic DNA. The primers were designed referring to the 5^′^ MGMT sequence annotated in UCSC sequence. The primer sequences are: MGMTgapF 5^′^-GAGGCAACCCAGACACTCAC-3^′^ and MGMTgapR 5^′^-CAGGACAGCCAGCGAGAC-3^′^. The purified PCR product (535 bp) was directly sequenced by the dye terminator method (PRIMM Facility, Naples, Italy). The sequence analysis showed that the gap was of 359 bp. This sequence was submitted to GenBank JX390728].

### DNA extraction from paraffin-embedded sarcoid samples, sodium bisulfite conversion and sequencing

Genomic DNA was isolated from paraffin-embedded sections using standard procedures (Strazzullo et al., 2003) and was precipitated with 250 mM NaCl and isopropanol. DNA extracted from paraffin embedded samples and cell lines (0.25 – 1 μg) was sodium bisulfite-converted using the EpiTect Bisulfite Kit (Qiagen, Germany), according to the protocol relative to the paraffin embedded samples. Samples were eluted in a final volume of 20 μl of elution buffer. To study the differential methylation status of MGMT putative promoter, bisulfite sequencing was performed. Briefly, 1 μl of sodium bisulfite-converted DNA was amplified using a double step PCR (semi-nested PCR) carried out using Gradient PCR Express (Hybaid, Middlesex, UK). The step 1 primer sequences are: MGMTbisF 5^′^-GAGGGAGGTAATTTAGATATTTAT-3^′^ and MGMTbisR2 5^′^-AAAAAACCTACAATAACAACAAC-3^′^; the step 2 primer sequences are: MGMTbisF and MGMTbisR2 5^′^-CACAAACAAACAAAAAAACCC-3^′^. The second amplification was carried out using 1 μl of first amplification product as template. The thermal cycling conditions were: 95°C for 3 min, followed by 95°C for 30 sec, 52°C for 45 sec and 72°C for 45 sec for 25 (first amplification) or 35 (second amplification) cycles. The product of the first amplification was of 628 bp; the second product was of 572 bp. After amplification, PCR products were recovered from agarose gel using QIAquick Gel Extraction Kit (Qiagen), following manufacturer’s instructions. Purified PCR products were cloned in the pGEMTeasy cloning vector (Promega corporation, USA). After cloning, DNA fragments were sequenced by the dye terminator method (PRIMM Facility, Naples, Italy).

## Competing interests

The authors declare that they have no competing interest.

## Authors’ contributions

GA and MS have contributed equally to this paper. AC and GA have contributed to experimental design and drafted the manuscript AC and FR have contributed to interpretation of data and have been involved in critically revising the manuscript GB has conceived the study, coordinated the group and drafted the manuscript MS has contributed to experimental design and drafted the manuscript RF has contributed to performing experimental procedures MDE has coordinated the group and drafted the manuscript.
